# Neurocosmetics or Hype? Psychobiotic Potential of Strain-Specific Cosmeceuticals

**DOI:** 10.3390/nu18050817

**Published:** 2026-03-02

**Authors:** Alexandra-Eleftheria Menni, Helen Theodorou, Georgios Tzikos, George Stavrou, Ioannis M. Theodorou, Eleni Semertzidou, Joanna Venieri, Aristeidis Ioannidis, Anne D. Shrewsbury, Katerina Kotzampassi

**Affiliations:** 1 Department of Surgery, Aristotle University of Thessaloniki, 54636 Thessaloniki, Greece; alexmenn@auth.gr (A.-E.M.); tzikos_giorgos@outlook.com (G.T.); ariioann@yahoo.gr (A.I.); a_shrewsbury@yahoo.com (A.D.S.); 2 Department of Sociology, School of Social Sciences, University of Crete, 74100 Rethymno, Greece; psy5057@psy.soc.uoc.gr; 3 Department of Surgery, 417 NIMTS (Army Share Fund Hospital), 11521 Athens, Greece; stavgd@gmail.com; 4 Hellenic Institute for the Study of Sepsis (HISS), 11528 Athens, Greece; itheodorou@med.uoa.gr; 5 Library of AHEPA University Hospital, 54636 Thessaloniki, Greece; elenisemer@gmail.com; 6 Scientific & Biomedical Information Centre, Storm Memorial Library, Windsor, IL 61957, USA; joannavenieri@yahoo.com

**Keywords:** cosmeceuticals, neurocosmetics, probiotics, *L. lactis* subsp. *cremoris* H61, *L. reuteri* DSM 17938, *W. coagulans* MTCC 5856

## Abstract

**Background:** There is increasing interest in cosmeceuticals—cosmetic regimes incorporating a specific probiotic or postbiotic strain, fully characterized genetically and phenotypically—which, when topically applied, have the ability to modulate the skin microbiome, exhibit anti-inflammatory properties and improve the overall skin appearance by reducing signs of aging. In addition, claims have been made that emotional and psychological well-being can be improved by neuroactive substances released by the probiotics in cosmeceuticals, acting via the skin–brain axis. However, claims are somewhat generalized and imprecise, and we deemed it important to look more precisely at published research relating to cosmeceuticals. There have been very few research publications on these products, identified as neurocosmetics, and they immediately provoked strong reactions from dermatologists and psychiatrists, mainly with regard to the ethical and safety aspects of their use. **Objectives/Method:** The present strain-centered literature evaluation aimed to select from peer-reviewed publications referring to cosmeceuticals only those dealing with fully characterized, specific probiotic strains with documented beneficial skin properties. Eligible strains found were subsequently subjected to a secondary search to ascertain whether they also demonstrated clinical, or even experimental, evidence of strain-specific psychobiotic properties. **Results:** From 33 strain-specific cosmeceuticals identified, only three strains—*Lactococcus lactis* subsp. *cremoris* H61, *Limosilactobacillus reuteri* DSM 17938, and *Weizmannia coagulans* MTCC 5856—demonstrated reproducible evidence of psychobiotic potential. **Conclusions:** Current evidence does not support the notion that cosmeceuticals are likely to directly modulate emotional states through topical application, since the coexistence of cosmeceutical and psychobiotic properties within the same probiotic strain seems to be both uncommon and highly strain-specific and therefore of little practical, generalized use.

## 1. Introduction

The role of cosmetic use—dating back to Queen Nefertiti of the Egyptians around 1300 BC—has been related to enhancing self-esteem and confidence and providing an element of control and self-care and subsequently increasing an individual’s feelings of capability, competence, dominance, social prestige and attractiveness, not least through the rituals of self-care and self-expression [1].

The US Federal Food, Drug, and Cosmetic Act defines cosmetics as “*articles intended to be…applied to the human body…for beautifying*, *promoting attractiveness*, *or altering the appearance*” [FD&C Act, sec. 201(i)]. This definition was further clarified by the Modernization of Cosmetics Regulation Act of 2022 (MoCRA) and modified to include “*a preparation of cosmetic ingredients with a qualitatively and quantitatively set composition for use in a finished product*” [FD&C Act, sec. 361]. The European Regulation No 1223/2009 defines cosmetics as “*any substance or mixture intended to be placed in contact with the external parts of the human body…with a view exclusively or mainly to cleaning*, *perfuming*, *changing their appearance*, *protecting them*, *keeping them in good condition or correcting body odors*”. Cosmetics, therefore, by definition, only affect the skin, or skin appendages. The same regulation [improving the 1976 Directive 76/768/EC] strengthens the safety of cosmetic products by the introduction of the notion of “*responsible person*” and includes the obligation to report serious undesirable effects. Additionally, EU No 655/2013 underlines that “*the safety of a cosmetic product must be demonstrated and data on the microbiological quality must be included*” in the Cosmetic Product Safety Report.

The skin is a dynamic organ that closely interacts with a complex ecosystem of microorganisms, known as the skin microbiota [2,3]. Often referred to as the “fourth skin layer”, the skin’s microbiota plays a critical role in skin maintenance and inflammation regulation. Recent clinical research focusing on microbiome restoration, by means of probiotics or postbiotics, has highlighted their potential to improve skin health and appearance [texture, lines, redness, aging] and to increase resilience [3]. Thus, in recent years, there has been a growing trend towards cosmetics containing live or non-viable [inanimate] probiotic bacteria, their cell wall fragments, or their metabolites—collectively named postbiotics—for reducing inflammation, enhancing immunomodulation and the epithelial barrier function and improving skin hydration, elasticity, and overall appearance, as well as reducing signs of aging, such as pigmentation, wrinkles and fine lines [4,5,6]. The new term ‘cosmeceuticals’ has thus been adopted to describe the very special category of cosmetics that can bridge the gap between drugs and cosmetics by incorporating bioactive ingredients claimed to have ‘medical’ benefits—exerting a pharmaceutical, but not necessarily a biological, therapeutic benefit [7].

However, there are “strictly” defined criteria for a cosmetic to be called a ‘cosmeceutical’: it must comply with regulations for the characterization of a bacterium as a specific probiotic strain, both genetically and phenotypically documented. Peer-reviewed publications of the experiments defining the rationale of their intended use are also required. Furthermore, the probiotic in the commercial product must have the same quantity of bacteria used in the clinical trials to benefit the designated target site, and, when humans are the intended receivers, the delivery technique, dosage, and length of use should have been determined through human studies [5,8,9,10]. In a fairly recent [mid-2024] review by our group [8], aimed at selecting from a high volume of publications only the clinical studies fulfilling the above prerequisites, a total of only 14 papers were found: five referring to cosmetics incorporating probiotics and nine to cosmetics incorporating postbiotics. These numbers contrast with the publication by Yadav et al. [11] in early 2023, which reported that as many as 928 patents had been retrieved from two databases [the CAS SciFinder and The Lens] after searches using the terms “probiotic” and “cosmetic” in only the previous 3 years and were mainly registered in the US, the Republic of China, and Korea.

Recently, another term, “neurocosmetics”, has been revived by Haykal and co-authors [12] and cautiously come to the forefront. Although used since the 1990s, it was defined in 2001 as “*products that are supposed to modulate the neuro-immuno-cutaneous system functioning at the epidermal level*” [13] and has also been defined as “*a transdisciplinary domain that investigates and formulates topical products with the intent to interact with the skin’s neurosensory system*, *modulate neuroimmune signaling*, *and influence psychophysiologic states*” or as “*a novel class of topical agents designed to act upon the skin’s neurosensory system and influence psychophysiological responses*, *thereby enhancing dermatologic function and emotional well-being*” [14,15]. Despite these previous references, the publication by Haykal and co-authors [12] raised an immediate and strong reaction in the form of published comments by dermatologists, psychiatrists, and psychologists on both the logic of the authors’ argument and the ethical aspect of the use of such preparations [16,17]. The foundation of the claim for neurocosmetic treatment is the skin–brain axis, a complex regulatory, bidirectional communication network. The skin contains a variety of neurotransmitter receptors and neuromediators, existing not only in the cutaneous nerve endings but also produced by keratinocytes, melanocytes, and immune cells—the local neuroendocrine system [18]. Interactions between cutaneous nerves, released neuropeptides, and barrier components mean that the skin influences and is influenced by emotional stress, neuroinflammation, and microbial dysbiosis, while the external, topical application of certain molecules has been found to reduce stress-related skin reactivity, thus contributing to visible improvements and psychological relief [19,20]. In other words, the argument is that the skin’s microbial diversity itself or the modulatory efforts of probiotics not only affect the skin barrier integrity and inflammatory state but may also influence emotional states via the gut–skin–brain axis [21,22].

However, such properties are mainly attributed to psychobiotics, a specific group of probiotics, the name standing for probiotics that confer mental health benefits when taken (at present) orally. They have the unique characteristic of producing and delivering neuroactive substances, which act on the brain–gut axis [23,24,25], and generally exert beneficial effects on stress, anxiety, depression, insomnia, and other psychopathological disorders by modifying/reducing the abundance of the disturbed microbiome [26,27,28].

Although this area holds considerable promise, it should be noted that knowledge of the neurocosmetic mechanisms involved in the restoration of the microbiota diversity, its relation with emotion regulation or stress-induced emotional dysregulation, and therefore its relation with dermatological ‘diseases’, is still at a very early stage. Most of the data are preclinical or tested only on low numbers of participants, often without controls, while the documentation of their properties is still somewhat subjective. According to current knowledge, the effect of topically applied cosmeceuticals regarding the regeneration and rebalance of aging skin is accelerated by the probiotics and/or their derivatives [8]. For this ‘cosmetic’ to simultaneously achieve emotional improvements, it must also exhibit psychotropic properties, that is, topically release neuropeptides and neuromodulators [23]. This whole subject, however, is relatively new and imprecise and lacks the support of rigorous research.

Thus, the aim of this targeted, strain-centered literature evaluation was to select from the volume of peer-reviewed publications referring to cosmeceuticals only those studies dealing with specific probiotic strains, live or inanimate [postbiotic], fully genetically and phenotypically characterized, and with their specific properties well documented and, furthermore, only those known to exert psychotropic properties. In other words, to find in the current, peer-reviewed literature the cosmeceuticals containing specific probiotics, which might, therefore, have not only cosmetic but also psychotropic properties.

## 2. Materials and Methods

### Search Strategies

A comprehensive literature search was performed in the PubMed and Scopus databases to identify all relevant articles on probiotic strains with documented properties in cosmeceutical contexts. Search terms included: cosmeceuticals, cosmetics, probiotics, postbiotics, skin or dermal, topical use or application, randomized clinical trials [RCTs], and clinical studies. Two authors [M.A.-E. and S.G.] with a research background in probiotics, working independently, initially searched the collected articles by title to exclude duplicates and those, by title and abstract, not relevant to the topic. They then went through the full texts of the remaining publications to identify and reject those articles not fulfilling the inclusion criteria. In a case of discrepancy or disagreement, a third author [K.K.] was involved to resolve the issue.

The studies finally used were only those which referred to cosmetic products [creams, emulsions, lotions, shampoos] containing probiotics or postbiotics [i] of a clearly identifiable strain [either single or multiple strains] that was fully genetically and phenotypically characterized; [ii] topically applied to healthy-skin volunteers; [iii] published after peer review; and [iv] with relevance to cosmetic endpoints. Articles referring solely to genus- or species-level effects without clear strain designation were excluded.

Each eligible strain found in these finally selected articles was then subjected to an independent secondary literature search, in PubMed and Scopus databases, to assess evidence of psychobiotic properties. This search focused on identifying experimental or clinical studies reporting effects on mental-health-related outcomes, including, but not limited to, depression-like or anxiety-like behaviors, stress responsivity, mood regulation, pain perception, and gut–brain axis modulation. Thus, search terms combined the full strain name paired with the terms psychobiotics, OR serotonin, OR dopamine, OR neuropeptides, OR GABA, OR mood, OR emotions, OR depression, OR anxiety, OR stress, OR pain, one by one. All relevant literature found was used for the present review.

## 3. Results

### 3.1. Identification of Strain-Specific Cosmeceutical Probiotics

Our search strategy in the PubMed and Scopus databases, covering up to December 2025 and focusing on probiotic or postbiotic strains with documented properties in cosmeceutical contexts, yielded 1065 records. After removing duplicates [*n* = 322] 743 records were screened by reading the titles and abstracts, of which 512 were considered irrelevant to the research. The remaining 231 studies were read in full, and a substantial number [*n* = 175] were excluded since they reported effects at the genus or species level only. This left 56 articles meeting the criteria for data extraction. From these 56 articles, a list containing the strains referred to [*n* = 33]—each specific strain tabulated only once—was compiled for further evaluation [Supplementary Figure—FlowChart].

### 3.2. Identification of Strain-Specific Cosmeceutical Probiotics with Psychobiotic Properties

Each one of the 33 strains identified from the cosmeceutical literature [56 articles] was subsequently subjected to an independent search in the PubMed and Scopus databases to assess the presence of psychobiotic-related properties. This secondary screening revealed that the majority of strains with cosmeceutical relevance lacked any published data linking them to mood-related outcomes, stress modulation, or gut–brain axis mechanisms and were, therefore, excluded from further analysis. Only three probiotic strains, in a total of 10 publications, demonstrated evidence of potential psychobiotic relevance, either through experiments on rodents or through preclinical studies or clinical investigations reporting psychological or stress-related outcomes. These three were: *Lactococcus lactis* subsp. *cremoris* H61, *Limosilactobacillus reuteri* DSM 17938 and *Weizmannia coagulans* MTCC 5856.

However, it should be mentioned from the beginning that the psychobiotic effects referred to in the studies and relating to these three strains were reached after oral administration, and no study deals with topical application [Table 1 and Table 2].

#### 3.2.1. *Lactococcus lactis* subsp. *cremoris* H61

*Lactococcus lactis* H61, or *Lactococcus lactis* subsp. *cremoris* H61, was one of the first probiotics established as a cosmeceutical and has also been identified as exerting psychotropic actions. There are four publications from the same group of investigators, referring to an ability to improve certain skin properties. Oral administration of heat-killed *L. lactis* H61 to aged SAMP6 mice was found to have the potential to suppress skin ulcers and to reduce hair loss, manifestations associated with aging in mice [29]. When the same heat-killed strain or placebo was given daily to 30 women, it led to a significant improvement in self-assessed skin elasticity at week 8 in relation both to baseline and the placebo [30]. Furthermore, 23 young females were orally given either *L. lactis* H61-fermented milk [10^10^ cfu per day] or conventional yogurt [*L. delbrueckii* ssp. *bulgaricus* and *S. thermophilus*] as a placebo daily for 4 weeks. Both treatments offered similar beneficial effects on skin status, but *L. lactis* H61 also showed an increase in sebum content in the cheek, and since skin lipids contribute to better maintenance of the skin barrier, the authors considered *L. lactis* H61 superior to yogurt [31]. They then tried the topical application of this strain as a cosmetic, consisting of the bacterial cell extract obtained with an aqueous solvent, and found that it could protect normal human epidermal keratinocytes from UVB damage by means of suppression of UVB-induced interleukin-8 production, compared to controls [32].

In 2025, researchers from the same institution in Japan tested the possible psychotropic effects of *L. lactis* H61 on environmentally restrained rats—exhibiting prolonged maladaptive immobility in the forced swim test, this being a recognized potential risk factor for depression. The postbiotic *L. lactis H61* seemed to alleviate the stress-induced depression, and a patent on the antidepressant effects of Lactococcus lactis H61 has been applied for [33]. This patent application is further supported by the authors’ reference to their studies on the behavioral and physiological stress responses of rats under environmental and chronic mild stress and exposed to multiple ‘environments’ designed to reduce the comfort of their living conditions—hunger, thirst, or direct physical distress excluded—while receiving heat-killed *L. lactis* H61 or a placebo for 8 weeks. The complementary effect of *L. lactis* H61 seemed to facilitate stress resilience without any added physiological burden, such as body weight, food intake, or estrous cycle [34].

#### 3.2.2. *Limosilactobacillus reuteri* DSM 17938

The oral consumption of yogurt containing *L. reuteri* strains, which has a long history as a “cosmetic”, established in homemade recipes as achieving radiant skin and hair in females, has also been further documented in mice as producing a thick lustrous coat, characterized as a ‘glow of health’ [35]. Furthermore, it seems to be related to the successful healing of skin wounds—more than twice as fast as in control animals [36].

These early observations led university-affiliated researchers related to the cosmetic industry to test a specific probiotic strain, *L. reuteri* DSM 17938—both in live form and as lysate—in an ex vivo ultraviolet B radiation-induced inflammation skin model, UV-B radiation once being considered the greatest promoter of extrinsic aging [37]. Their results revealed that both the live bacteria and the lysate, or so-called postbiotic—prepared after mechanical high-pressure homogenization—evidenced anti-inflammatory activity able to prevent photo-aging and improve skin barrier function, along with an inhibitory action against skin pathogens. Following this, the safety and efficacy of live *L. reuteri* DSM 17938 [1 × 10^8^ cfu/gr] or placebo, incorporated in a well-known ointment based on shea butter and canola oil, typically used for treatment of various inflammatory skin conditions, was tested on 34 humans for a 2-month period. The appraisal by the participants as to the cosmetic acceptability and efficacy was found comparable between groups, while the SCORing Atopic Dermatitis index [SCORAD]—a clinical tool for objectively assessing the total area involved, the intensity of the lesions, and the subjective symptoms of itching and sleep loss—showed a tendency towards reduction compared to controls [38].

Regarding psychotropic properties, *L. reuteri* DSM 17938 was initially tested in the newborn mouse model of maternal separation stress [39]. Placebo-treated mice, unpredictably separated from their mothers for postnatal days 5 to 14, were significantly less vocal due to stress in relation to those treated with *L. reuteri*. Furthermore, those treated but not separated had markedly increased vocalization in relation to the placebo. *L. reuteri* DSM 17938, which up-regulates opioid peptides, kappa-opioid receptor-1 genes and CD200 and down-regulates the chemokine receptor CCR2 transcripts in the stressed brain, was found to modulate cerebral genes related to pain and stress reduction and anti-inflammatory signaling [39]. The investigators, continuing with the same model, then reported that *L. reuteri*-treated mice, although separated from the mother, demonstrated better cognitive function and less anxious behavior, directly related to the reduction in increased corticosterone and Glial Fibrillary Acidic Protein [GFAP] brain levels—a biomarker of neural damage—and to a reduction in both Cholecystokinin [CCK] and early postnatal growth, due to stress [40].

Furthermore, Mazzoli A, et al. [41] investigated the inhibiting effects of *L. reuteri* DSM 17938, given to rats at a dose of 10^8^ cfu/0.5 mL, in counteracting high-fat and high-fructose diet-induced neuroinflammation, endoplasmic reticulum stress, and autophagy in the hippocampus, an area involved in learning and memory. The neuroprotective action is attributed to the ability of the probiotic to down-regulate Western diet-induced metabolic endotoxemia and systemic inflammation. In another mouse model of lipopolysaccharide [LPS]-induced depression and anxiety-like behaviors, *L. reuteri* DSM 17938 [5 × 10^9^ cfu/mL] significantly reversed behavior phenomena, such as reduced distance of movement, center zone stay time and immobility time in the forced swimming test. In parallel, it restored gut microbial diversity, intestinal metabolic pathways, including amino acid and unsaturated fatty acid metabolism pathways, and neural homeostasis [42].

Two similar RCTs—from China and India—were conducted on the effectiveness of *L. reuteri* DSM 17938 [10^8^ cfu per day] in reducing infantile colic and, consequently, maternal depression. Treatment versus placebo, applied for 21 days to breastfed infants under 4 months of age, showed a significant reduction in crying time, which might be associated with an improvement in maternal depression [Edinburgh postnatal depression scale], unanticipated because the mothers received no treatment [43,44]. In a clinical trial initially aiming to investigate whether the long-term administration of *L. reuteri* DSM 17938 [4 × 10^8^ cfu per day for 15 days and then 2 × 10^8^ cfu per day up to the 105th day] could ameliorate functional constipation through specific gastrointestinal peptide pathways, Riezzo et al. [45] found that both 5-HT and BDNF significantly decreased in relation to baseline values in *L. reuteri*-treated participants. This finding strongly supports its psychotropic effects, along with the alleviation of functional constipation.

Finally, in a recent clinical study [46], the authors aimed to investigate the mechanisms underlying the putative antidepressant effects of probiotics—hypothesizing that they are mediated via short-chain fatty acids [SCFAs]—in a population suffering ‘inflammatory depression’, characterized by obesity [BMI ≥ 25 kg/m^2^ and hs-CRP ≥ 1 mg/L, indicating systemic low-grade inflammation] and a lower response to conventional antidepressants. A combined formula of *L. reuteri* DSM17938 and *L. reuteri* ATCC PTA 6475 in a dose of 2 × 10^8^ cfu/tablet was administered twice daily as an add-on to antidepressant treatment for 8 weeks against the placebo. In general, there was no statistically significant difference between groups, either in depression or inflammatory scores or in SCFAs levels in blood and feces between baseline and week 8, as well as no significant correlation between the inflammatory score and depression severity. However, among the SCFAs analyzed, only the formic acid changes in feces gave a significant correlation with the depression score [MADRS] over 8 weeks of probiotic treatment. In detail, the baseline formic acid was found significantly correlated [r = 0.43, *p* = 0.008] with the changes in MADRS, indicating that a low formic acid value at baseline was associated with a greater antidepressant effect, while a greater increase in formic acid at week 8 was associated with a superior treatment response [r = −0.45, *p* = 0.009] [46].

#### 3.2.3. *Weizmannia coagulans* MTCC 5856

*Weizmannia coagulans,* first described in 2020, was formerly recognized as *Bacillus coagulans* [47], having the characteristics of both *Lactobacilli*—producing lactic acid—and *Bacilli*, due to its ability to form spores. Several strains have been shown to play a potential role in the modulation of microbiota composition and activity, enhancement of immunity, alleviation of metabolic disorders [type-2 diabetes and non-alcoholic fatty liver disease], and to exhibit anti-inflammatory effects, all characteristics being strain-specific [48,49,50].

In particular, the strain *W. coagulans* MTCC 5856 has also been reported to alleviate acne and reduce skin aging in healthy females and is thus included in cosmeceuticals. In the form of LactoSporin^®^ [Sami-Sabinsa Group Ltd., Bangalore, India]—an extracellular metabolite purified from *W. coagulans* MTCC 5856 fermented broth with an INCI name *Bacillus* ferment filtrate extract—a 2% LactoSporin^®^ cream applied for 10 weeks was found to significantly reduce wrinkles and crow’s feet, nasolabial folds, frown lines, and fine facial lines and improve skin elasticity, hydration and texture compared to both baseline dermatological assessment and placebo treatment [51,52].

In addition, the same strain was also found to ameliorate or prevent major depressive disorders in 40 irritable-bowel-syndrome patients, randomized to receive either placebo or *W. coagulans* MTCC 5856 at a dose of 2 × 10^9^ cfu per day, daily for 3 months. The before–after assessment of participants, by means of psychometric questionnaires [Hamilton Rating Scale for Depression, Montgomery–Asberg Depression Rating Scale and Center for Epidemiological Studies Depression Scale], in parallel with the irritable bowel syndrome quality of life questionnaire, revealed significant improvement [*p* = 0.01] in major depressive disorders, making it an important innovative treatment option in IBS patients [53]. This finding is probably closely related to the reduction of abdominal distension in such patients. On the other hand, another strain, the Unique IS-2, is known to alleviate anxiety-like behaviors by up-regulating hippocampal BDNF [48], and the BC99 strain by regulating the level of neurotransmitters and/or the change in gut microbiota [54].

**Table 1:** Εxperimental and clinical evidence supporting the psychotropic effects of administered probiotic strains having cosmetic properties.

Study Type	Model	Route	Dose	Duration	Comparator	Psychotropic Outcomes	Ref.
*Lactococcus lactis* subsp. cremoris H61							
Experimental (rats)	Environmental restrained stress	Oral	Diet + 0.05% (*w*/*w*) heat-killed H61	8 weeks	Standard diet	reduces depression-like behavior improves intestinal microbiota profile	[33]
Experimental (female rats)	Environmental chronic mild stress	Oral	Diet + 0.05% (*w*/*w*) heat-killed H61	8 weeks	Standard diet	reduces anxiety-like behaviors enables stress resilience no effect on body weight, food intake, estrous cycle, and sociability	[34]
Experimental (newborn mice)	Maternal separation stress	Oral gavage	10^6^ cfu/day in PBS 100 μL	10 days	PBS 100 μL	increases vocalizations in mice allowed to stay with their dams. modulates brain genes [related to stress and pain] down-regulates CCR2 transcripts, in the stressed neonatal brain	[39]
*Limosilactobacillus reuteri* DSM 17938							
Experimental (newborn mice)	Unpredictable maternal separation stress	Oral gavage	10^6^ cfu/gr BW/day in PBS 100 μL	14 days	PBS 100 μL	improves spatial memory and cognitive function ameliorates anxious behavior reduces stress-associated proteins in the brain modulates gut microbial dysbiosis ameliorates weight loss	[40]
Experimental (rats)	High-fat/high-fructose diet–induced neuro-inflammation	Oral	10^8^ cfu/0.5 mL 10% sucrose	8 weeks	0.5 mL 10% sucrose	neuroprotection reduces hippocampal neuroinflammation reduces endoplasmic reticulum stress reduces autophagy preserves neuronal plasticity-related proteins	[41]
Experimental (mice)	LPS-induced depression and anxiety	Oral gavage	5 × 10^9^ cfu/mL	10 days	Fluoxetine [20 mg/kg, i.p.]	reduces LPS-induced depressive- and anxiety-like behaviors restores gut microbial diversity improves intestinal metabolic functions restores amino acid metabolism-related pathways in hippocampus and prefrontal cortex	[42]
Clinical (breastfed infants)	Infantile colic; colicky-induced maternal depression	Oral	10^8^ cfu/day	21 days	Identical formulation	reduces infant crying time improves maternal depression	[43]
Clinical (infants < 5 mo)	Infantile colic; colicky-induced maternal depression	Oral	10^8^ cfu/5 drops once daily	21 days	Observatio-nal study [compared to baseline]	78.9% reduction of infant crying time from baseline 63% reduction of maternal depression from baseline	[44]
Clinical (adults)	Functional constipation	Oral	4 × 10^8^ cfu/d for 15 d; then 2 × 10^8^ cfu/d twice daily up to day 105	105 days	Identical formulation	reduces 5-HT serum levels in relation to baseline reduces BDNF serum levels in relation to baseline	[45]
Clinical (under antide pressant treatment)	MDD + obesity (BMI ≥ 25 kg/m^2^; hs-CRP ≥ 1 mg/L)	Oral [add-on]	2 × 10^8^ cfu/tb twice a day [each tb also contains the strain ATCC PTA 6475]	8 weeks	Identical formulation	no significant antidepressant effect (MADRS and PHQ-9) no difference in inflammatory scores no difference in total SCFAs increases of fecal formic acid at week 8 well correlated to treatment response (MADRS improvement)	[46]
*Weizmannia coagulans* MTCC 5856							
Clinical	IBS patients with MDD	Oral	2 × 10^9^ cfu/d	90 days	Identical formulation	reduces serum myeloperoxidase in relation to baseline improves depression scores (HAM-D, MADRS, CES-D) improves IBS-QOL	[53]
Clinical	Depression or Anxiety behavior	Oral	5 × 10^9^ cfu/d in 3 g dextrin	8 weeks	dextrin (3 g/day)	improves depression scores in relation to placebo reduces inflammatory cytokines IL-17 and IL-10, increases neurotransmitter levels of γ-GABA and NO restores microbial diversity increases the production of SCFAs	[54]

PBS: phosphate buffered saline; BW: body weight; IBS: Irritable bowel syndrome; MDD: major depressive disorder.

**Table 2:** Comparison of the three identified cosmeceuticals, regarding their evidence as psychobiotics.

**Strain**	Cosmetic Evidence			Psychobiotic Evidence		
	Type of Study [Ref.]	Route of Administration	Skin Effects	Type of Study [Ref.]	Route of Administration	Brain/Gut Effects
***Lactococcus lactis*** **subsp. *cremoris H61***	Experiment Mice [29]	oral	Skin anti-aging	Experiment Rats [33]	Oral	reduces depression-like behavior improves gut microbiota dysbiosis
	Clinical Woman [30]	Oral heat-killed	Improved skin elasticity	Experiment Rats [34]	Oral	reduces anxiety-like behaviors enables stress resilience no effect on body weight, food intake, estrous cycle
	Clinical Woman [31]	Oral fermented milk	Increased sebum content and serum oxidative status			
	Ex vivo UVB-damage [32]	Topical heat-killed	Protect the irradiated human epidermal keratinocytes			
***Limosilactobacillus reuteri DSM 17938***	Experiment Aged Mice [35]	oral	increased subcuticular folliculogenesis lustrous hair	Experiment Newborn Mice Maternal separation stress [39,40]	Oral gavage	modulates brain genes [related to stress and pain] improves spatial memory and cognitive function ameliorates anxious behavior modulates gut dysbiosis
	Experiment Mice skin wound [36]	oral	faster wound-healing through up-regulation of oxytocin	Experiment Rats diet-induced neuroinflammation [41]	oral	reduces hippocampal neuroinflammation, endoplasmic reticulum stress and autophagy preserves neuronal plasticity
	Ex vivo and in vitro UVB-damage [37]	Topical live bacteria and lysate	anti-inflammatory reconstructs hu-man epidermis, enhances skin barrier against skin pathogens	Experimental Mice LPS-induced depression and anxiety [42]	oral compared to Fluoxetine	reduces depressive- and anxiety-like behaviors restores gut diversity improves gut metabolic functions restores amino acid meta-bolism in hippocampus and prefrontal cortex
	clinical [38]	topical	reduces intensity of lesions, and itching	Clinical Infantile colic maternal depression [43,44]	oral	reduces infant crying time improves maternal depression
				Clinical Functional constipation [45]	oral	reduces 5-HT and BDNF serum levels
				Clinical MDD + obesity antidepressants treatment [46]	oral [add-on]	no effect on depression, inflammatory scores, and total SCFAs increases of fecal formic acid well-correlated to treatment response
***Weizmannia coagulans MTCC 5856***	clinical moderate acne [51]	Topical LactoSporine^®^ cream	reduces acne severity and sebaceous secretion	Clinical IBS patients with MDD [53]	oral	reduces serum myelo-peroxidase in relation to baseline improves depression score (HAM-D, MADRS, CESD) improves IBS-QOL
	clinical [52]	Topical Lacto-Sporine^®^ cream	reduces wrinkles, and skin fine lines improves skin elasticity and hydration	Clinical Depression or Anxiety behavior [54]	oral	improves depression reduces inflammation increases γ-GABA and NO restores microbial diversity increases production of SCFAs

## 4. Discussion

The results of the present study, which focused on the literature search for specialized probiotics presenting psychotropic properties, namely psychobiotics, among the volume of peer-reviewed publications referring to cosmeceuticals, identified only three psychobiotic strains among the 33 cosmeceuticals found, with the prerequisite that they be live or inanimate, fully genetically and phenotypically characterized probiotic strains [7,8]. This study used a targeted, strain-centered literature evaluation, aiming to investigate the overlap between the specific probiotic strains used in cosmeceutical applications and their potential psychobiotic properties. The methodological approach was intentionally non-systematic but hypothesis-driven and targeted to ensure strain specificity and mechanistic relevance rather than an exhaustive coverage of all probiotic studies. Its conceptual framework was partly motivated by the emerging dialogue on neurocosmetics and emotionally intelligent skincare, triggered by the publication of Haykal et al. [12], who hypothesized an expanded use of cosmetics containing probiotic formulas as likely to lead to mood modification through the skin–brain axis. This viewpoint suggests that proper probiotic cosmetics, i.e., cosmeceuticals, may be implicated not only in skin physiology, stimulating anti-aging properties, but also in emotional and psychological states, through the neuro-cutaneous signaling pathways, since the skin–brain axis is now well-accepted, due to their common embryonic origin in the ectoderm [16,55]. This proposal, although at first glance appearing innovative, is, in fact, provocative and principally contrary to nature.

It has been correctly recognized that the skin is part of a continuous, bidirectional and complex regulatory network to the brain, through which it can influence and be influenced by emotional stress, neuroinflammation, and microbial dysbiosis [12,16,19,21]. Chronic social stress, anxiety, and other emotional issues can trigger skin inflammation via neuro-immune pathways and by changing microbial diversity, both in the gut and on the skin, and can thus exacerbate dermatological conditions, such as acne, eczema, and psoriasis [16,56]. Healthy skin can boost self-confidence and reduce stress; furthermore, if facial and neck skin is clear, glowing, fully hydrated, well-groomed, and wrinkle-free and carefully made up by cosmetic toners, serums, and moisturizer creams supported by foundation, blushers, eyelashes, mascaras and lipsticks, it may act to enhance one’s self-image [57]. The use of beauty products, regardless of their origin, ingredients, manufacturers, and cost, is nowadays an integral part of daily care for many throughout the world, as well as a kind of psychotherapy [58,59,60,61], since it is motivated by the human desire to be attractive, fight the signs of age, and tone down physical damage [8,62,63,64]. All these cosmetics, whether reinforced with specific probiotics or not, cannot be said to locally produce neurotransmitters or similarly communicate positive emotions to the brain [16].

Thus, the opinion of Haykal et al. [12], initially referred to, has rightly been met with prompt and rigorous criticism [13,17], emphasizing that cosmeceuticals are, by definition, a very special category of cosmetics that can bridge the gap between cosmetics and drugs by incorporating bioactive ingredients considered to exert ‘medical’ benefits but are strictly confined to the skin. According to Choi et al. [7], they exert a pharmaceutical therapeutic benefit but not necessarily a ‘biological therapeutic benefit’. Subsequent criticism by Dedeepya et al. [17] and Misery et al. [13] underlines a fundamental concern or even a fear within this field: the risk of theoretical exaggeration and the tangles of cosmetic ‘medical’ effects with excessive, suspicious, or even unlawful psychotropic outcomes. Of course, the emotional impact of various interventions, such as massage or aromatherapy [14,65], or even the use of a simple lanolin cream that merely softens the skin without any subsequent aesthetic improvement, is well known and not questionable. Likewise, food and clothing can also provide pleasant sensations, yet there is no talk, to date, of neuro-foods or neuro-clothing [16], even when they affect hedonistic feelings [14]. However, they can accelerate individually perceived psychological feelings, from a completely indifferent emotion to an illusory state of bliss. In the same manner, cosmetic products are just pleasant cosmetics, not neurocosmetics, and nothing new is being offered [16].

In a recent review of ‘The Fascinating World of Skin–Brain Connection’, Rizzi et al. [14] clearly says that since the neuro-molecules synthesized in the brain are the same as those of the skin’s nervous system for cell-to-cell communication, their ability to change emotions ‘has been wrongly attributed to neurocosmetics’. However, these neuro-molecules exert different local functions: Rizzi et al. [14] present the cases of *Schinus terebinthifolia* and *Sacha inchi* extracts, which, when applied to the skin, increase the release of dopamine by skin neurons, leading to increased skin blood flow, enhancement of the skin barrier performance, and finally the global improvement of the epidermis [66,67], while brain-produced dopamine acts as a neurotransmitter involved in brain network complexes related to pleasure, satisfaction, and positive mood [68]. Only in a case of a massive cutaneous dopamine release could it enter the bloodstream and reach the brain, but this is not a probable scientific hypothesis for a cosmetic product, which, by definition, must not penetrate the skin but remain on the epidermis [14].

Returning to the findings of the present strain-centered evaluation, we consider that these findings can provide a clarifying perspective within this debate. So, instead of attributing psychotropic or emotional properties directly to a topically applied cosmetic, our results demonstrate that, to date, only a limited subset of the fully genetically and phenotypically characterized probiotic strains found in cosmeceuticals additionally provide independent, strain-specific evidence of psychobiotic activity. Importantly, however, it should be emphasized that these psychotropic or emotional activities—although supported by both experimental [33,34,39,40,41,42] and clinical studies [43,44,45,46,53], published after peer review—were conducted in a field outside the cosmetic domain, since the probiotic strains were primarily given orally and not topically applied. Thus, they work through the gut–brain axis circuit, and, as such, the psychobiotic properties identified and analyzed for the three specific strains, *Lactococcus lactis* subsp. *cremoris* H61, *Limosilactobacillus reuteri* DSM 17938 and *Weizmannia coagulans* MTCC 5856, cannot be ascribed to their cosmetic action per se but rather to the intrinsic biological characteristics of these specific probiotic strains.

The above distinction is critical, as it could harmonize the innovative concept of neurocosmetics with the concerns raised by those who pose counterarguments [12,15]. Our findings do not encourage the notion that cosmeceuticals with psychobiotic properties directly modulate emotions. In such a case, both ethical and safety problems would arise, since it is not inconceivable that uncontrolled repeated use could lead to addiction [17,69]. Instead, our findings, based on the literature to date, suggest that certain cosmeceutical probiotics could be said to contribute to emotional or stress-related outcomes only when considered within a broader, systemic context that extends beyond their topical application on the skin. In this sense, psychobiotic effects do not emerge as related to, or as a consequence of, their cosmetic properties but as distinct, biologically based properties, with thoroughly selected probiotic strains having proven relevance to the gut–brain axis as well.

Our strain-focused analysis emphasizes the huge gap that exists between the numerous probiotics and postbiotics used in cosmeceutical applications and the limited availability of psychobiotics among them. Despite the growing research interest in gut–skin–brain axis applications, the results show that only a small subset of cosmeceuticals also exhibits measurable psychobiotic properties, a finding that calls into question the hypothesis or even the wish that probiotic cosmetic strains could easily act as psychobiotics as well, improving the mood of users through their cosmetic actions [12]. Furthermore, only these three cosmeceutical-specific strains, which also exert psychobiotic properties when orally given, have completely distinct levels and types of evidence to support their properties. *L. lactis* subsp. *cremoris* H61 represents an emerging psychobiotic with consistent preclinical results when given orally, demonstrating stress resilience and antidepressant-like effects in rodent models of environmental and chronic mild stress. However, the absence of human data and the reliance only on experimental models currently limit their translational relevance [33,34]. In contrast, *L. reuteri* DSM 17938, orally given, exhibits the most comprehensive and diverse evidence base, covering models of early-life stress, diet-mediated neuroinflammation, immuno-neuroendocrine modulation, and multiple clinical contexts [39,40,41,42]. While the human studies suggest indirect psychotropic benefits—particularly infantile colic reduction through gut peptide signaling—psychological outcomes were often secondary findings, thus underlining the need for new trials with emotional endpoints as primary outcomes [43,44,45,46]. *W. coagulans* MTCC 5856, also given by mouth and not topically applied, stands out as the only strain of the three with direct clinical evidence for improvement of depressive symptoms, although these are comorbidities in an irritable bowel syndrome population, thus raising the possibility that mood improvement may partially be mediated through gastrointestinal symptom improvement [53]. Collectively, these strains prove that psychobiotic effects from the cosmeceutical probiotics are strain-specific and frequently act indirectly, reinforcing the necessity for cautious interpretation and targeted clinical validation rather than broad extrapolation across cosmetic probiotic formulations.

Despite the comprehensive screening and discriminatory demonstration of cosmetic probiotic strains also exhibiting psychobiotic effects, some limitations must be acknowledged. Mainly, the complete failure to find a study in which the probiotic strains contained in the cosmeceuticals manifested their psychotropic action when applied topically. This, while initially appearing to be a weakness of the research, ultimately constitutes a strong indication that psychobiotic action cannot be demonstrated by probiotic products applied to the skin. Of course, the small number of cosmetic products containing probiotics and reporting psychobiotic effects in our study (3 out of 33) limits the generalization of our findings. Additionally, data supporting psychobiotic properties are generally based on in vitro, experimental, and preclinical trials and a few small-scale clinical studies with short-term follow-up, while the methodology used is rather indirect—heterogeneous markers rather than strong clinical or laboratory data.

## 5. Conclusions

The findings of this review clearly indicate that the coexistence of cosmeceutical and psychobiotic activity in the same probiotic strain represents the exception rather than the rule. Only three fully characterized probiotic strains used topically as cosmeceuticals, when given orally, demonstrate documented, independent, reproducible evidence of psychotropic benefits, which are supported by experimental and/or clinical studies conducted outside the cosmetic setting. Importantly, their psychobiotic activity is linked to systemic biological pathways, mainly the gut–brain axis, and not to topical skin application, that is, the skin–brain axis. These findings do not justify the assumption that cosmeceuticals can directly modulate mood or emotional states but instead emphasize the importance of careful interpretation, clear separation between cosmetic and psychobiotic claims, and further targeted clinical research before such concepts can be safely and scientifically integrated into the new field of neurocosmetics. Therefore, any future discussion on neurocosmetics should be based on solid biological evidence, avoid broad extrapolations, and take into account both the limitations of topical cosmetic use and the requirements of safety and ethics.

## 6. Future Perspectives

Future perspectives in this field should focus on well-designed human studies with topical application of probiotic strains exhibiting both cosmetic and psychotropic properties—or even with probiotic regimes combining two or more probiotics with either action. These studies should include objective, quantifiable endpoints, such as validated psychometric scales combined with standardized biomarkers, such as salivary cortisol and neuropeptide profiling, rather than relying on subjective mood reports alone. The design of such rigorous studies could be facilitated by the use of AI regarding the identification of sub-groups according to their skin microbiome profiles to facilitate the more accurate probiotic strain selection, in a way analogous to current classifications of skin types in cosmetic science.

Future research should also investigate whether combo regimes of probiotic strains with cosmetic and psychotropic actions could be safely and effectively developed for topical application without overemphasizing either of their roles. More accurate and rigorous studies will also allow regulatory frameworks to be adapted to prevent misleading psychotropic claims for cosmetic products. Finally, a realistic integration of the concept of the gut–skin–brain axis may help advance neurocosmetics as a scientifically grounded field rather than a speculative one. Until then, the incorporation of non-biological factors that support emotional well-being, such as personalized fragrance, texture, or even packaging, all developed within a strict safety and regulatory framework, could provide enhancement of emotional well-being without confusing cosmetic use with psychoactive activity.

## Data Availability

No new data were created or analyzed in this study.

## Supplementary Materials

The following supporting information can be downloaded at: https://www.mdpi.com/article/10.3390/nu18050817/s1, Supplementary Figure—Flowchart: PRISMA-like flow chart illustrating the search steps relating to cosmeceuticals for identification and evaluation of probiotic strains with potential psychobiotic properties.
