# Supplementary material for: Neurocosmetics or Hype? Psychobiotic Potential of Strain-Specific Cosmeceuticals
Source: Nutrients. 2026 Mar 2;18(5):817. doi: 10.3390/nu18050817 (PMC12986703; doi:10.3390/nu18050817)
Supplement: Supplementary file 1 [file nutrients-18-00817-s001.zip › nutrients-4131968-supplementary.pdf]

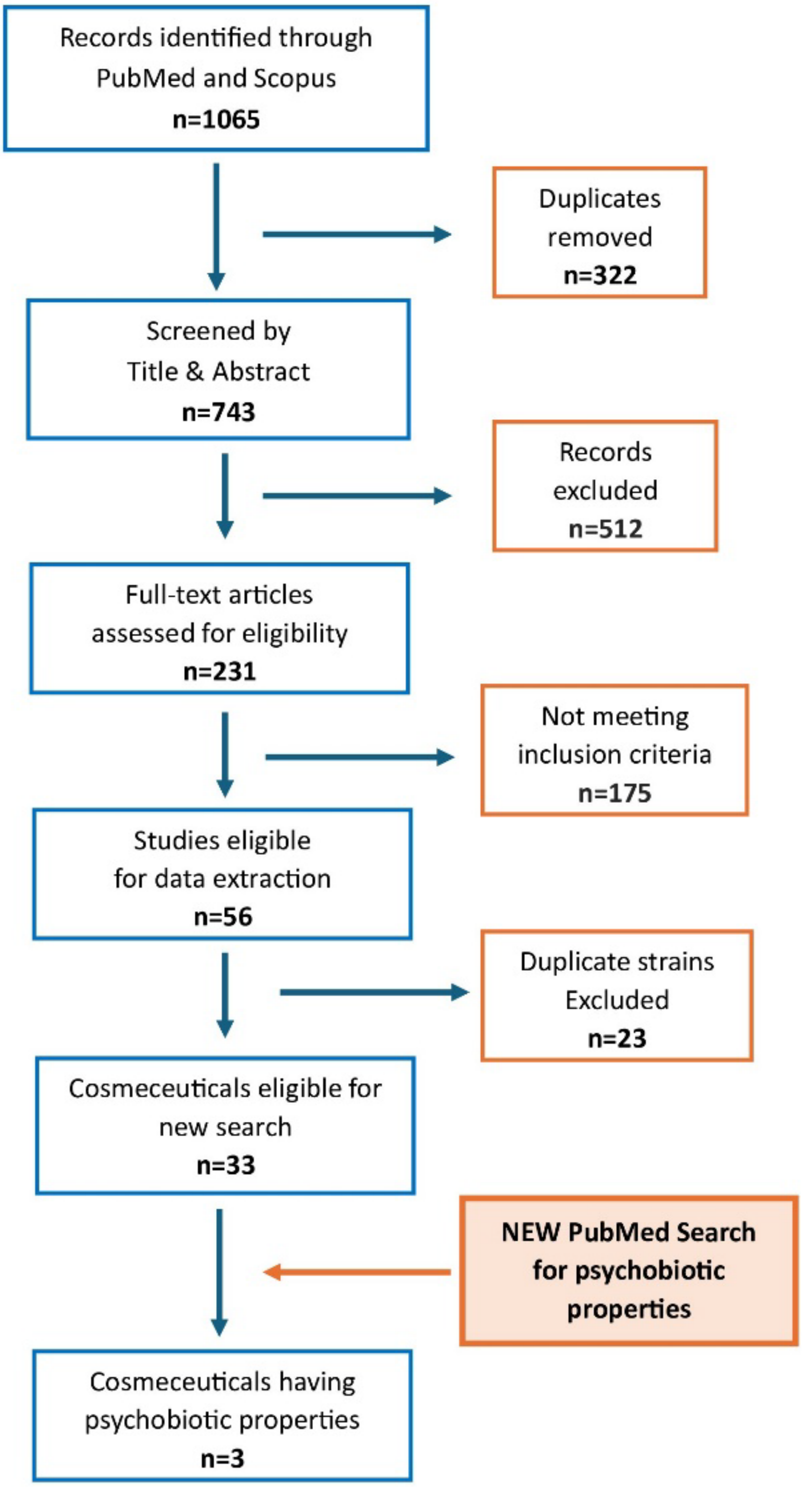


**Supplementary Figure – FlowChart:** PRISMA-like flow chart illustrating the search steps relating to cosmeceuticals for identification and evaluation of probiotic strains with potential psychobiotic properties.
